# Exocytosis and Endocytosis of Small Vesicles across the Plasma Membrane in *Saccharomyces cerevisiae*

**DOI:** 10.3390/membranes4030608

**Published:** 2014-09-03

**Authors:** Kathryn Stein, Hui-Ling Chiang

**Affiliations:** Department of Cellular and Molecular Physiology, Penn State College of Medicine, 500 University Drive, Hershey, PA 17033, USA; E-Mail: kstein@hmc.psu.edu

**Keywords:** fructose-1,6-bisphosphatase, phosphoenolpyruvate carboxykinase, isocitrate lyase, malate dehydrogenase, glyceraldehyde-3-phosphate dehydrogenase, cyclophilin A, vacuole import and degradation, extracellular vesicles, exosomes

## Abstract

When *Saccharomyces cerevisiae* is starved of glucose, the gluconeogenic enzymes fructose-1,6-bisphosphatase (FBPase), phosphoenolpyruvate carboxykinase, isocitrate lyase, and malate dehydrogenase, as well as the non-gluconeogenic enzymes glyceraldehyde-3-phosphate dehydrogenase and cyclophilin A, are secreted into the periplasm. In the extracellular fraction, these secreted proteins are associated with small vesicles that account for more than 90% of the total number of extracellular structures observed. When glucose is added to glucose-starved cells, FBPase is internalized and associated with clusters of small vesicles in the cytoplasm. Specifically, the internalization of FBPase results in the decline of FBPase and vesicles in the extracellular fraction and their appearance in the cytoplasm. The clearance of extracellular vesicles and vesicle-associated proteins from the extracellular fraction is dependent on the endocytosis gene *END3*. This internalization is regulated when cells are transferred from low to high glucose. It is rapidly occurring and is a high capacity process, as clusters of vesicles occupy 10%–20% of the total volume in the cytoplasm in glucose re-fed cells. FBPase internalization also requires the *VPS34* gene encoding PI3K. Following internalization, FBPase is delivered to the vacuole for degradation, whereas proteins that are not degraded may be recycled.

## 1. Introduction

*Saccharomyces cerevisiae* is an excellent model system to study cellular responses to environmental changes such as temperature, oxidative stress, and the availability of carbon sources [1,2,3,4,5,6,7,8,9,10,11,12,13,14,15]. Yeast cells can obtain energy through the utilization of sucrose, galactose, glucose, fructose, melibiose, and maltose [1,2,3,4,5,6]. Yeast cells also use non-fermentable carbon sources such as glycerol, pyruvate, acetate, and lactate to produce energy [1,2,3,4,5,6,16]. The addition of glucose to cells grown in non-fermentable carbon sources results in a rapid change in transcription [6,17]. An estimated 40% of genes in yeast alter their expression by more than two-fold within minutes after addition of glucose [17]. Glucose increases the expression of genes involved in ribosomal functions, glycolysis, and cell division [1,2,3,4,5,6,16,17,18,19,20,21,22,23,24,25,26,27]. Glucose also represses genes required for mitochondrial functions and genes encoding for gluconeogenic enzymes that include FBP1 (fructose-1,6-bisphosphatase), ICL1 (isocitrate lyase), PCK1 (phosphoenolpyruvate carboxykinase), and MLS1 (malate synthase) [4,6,17,26,28,29,30]. Likewise, glucose represses genes involved in the metabolism of sugars such as maltose and galactose. The repression of genes by glucose is known as “catabolite repression” [4,16,17,28,31,32,33].

Additionally, glucose causes changes in the turnover rates of mRNA and proteins. It decreases the turnover rates of mRNAs for the 40S and 60S ribosomal subunits [17,34], and increases the turnover rates of mRNAs for PCK1 and FBP1 [4,5,16,35,36,37]. At the protein level, glucose inactivates gluconeogenic enzymes through an increase in the rate of degradation. This is referred to as “catabolite inactivation” [38,39,40,41]. Catabolite inactivation has been observed in gluconeogenic enzymes that include fructose-1,6-bisphosphatase (FBPase), phosphoenolpyruvate carboxykinase (Pck1p), isocitrate lyase (Icl1p), and malate dehydrogenase (MDH2) [37,42,43,44,45,46,47,48,49]. Glucose also inactivates mitochondrial enzymes such as cytochrome c oxidase, aconitase, mitochondrial ATPase, and NADH dehydrogenase [35,50,51,52].

Gluconeogenic enzymes are induced with half-lives greater than 100 h when yeast cells are grown in media containing low glucose. However, when glucose is added to glucose-starved cells, these proteins are inactivated and degraded with half-lives of 20–40 min [45,49,53,54]. This inactivation and degradation of gluconeogenic enzymes during glucose re-feeding prevents energy futile cycles that may be detrimental to cells. The key gluconeogenic enzyme fructose-1,6-bisphosphatase (FBPase) has been studied extensively for catabolite inactivation. FBPase is either ubiquitinated and degraded in the proteasome [55,56], or phosphorylated and degraded in the vacuole [42,57,58,59,60]. The site of FBPase degradation is dependent on the duration of starvation [49]. When glucose is added to cells that are starved of glucose for 1 day, this protein is degraded in the proteasome [49]. In contrast, when glucose is added to cells that are starved for 3 days, FBPase is degraded in the vacuole [49]. For the vacuole-dependent pathway, the RAS2/PKA signaling pathway is activated leading to FBPase phosphorylation and subsequent degradation in the vacuole [58,59,60,61,62,63,64]. Other gluconeogenic enzymes that are degraded in the vacuole following glucose replenishment include malate dehydrogenase (MDH2), malate synthase (Mls1p), phosphoenolpyruvate carboxykinase (Pck1p), and isocitrate lyase (Icl1p) [45,49].

## 2. Small Vesicles Carry Gluconeogenic Enzymes to the Vacuole for Degradation via the Vacuole Import and Degradation Pathway

The vacuole import and degradation (Vid) pathway utilizes small vesicles to carry the gluconeogenic enzymes FBPase, MDH2, Pck1p, and Icl1p to the vacuole for degradation following glucose replenishment to glucose-starved cells. Mutants defective in the glucose-induced degradation of FBPase in the vacuole were isolated [54]. These mutants were classified into two groups based on FBPase distribution patterns determined by immunofluorescence microscopy [54]. Some mutants exhibited diffuse FBPase staining, while others displayed FBPase staining in punctate structures [54]. Distribution of FBPase in punctate structures suggests that FBPase is associated with membranous structures. Using S-1000 size chromatography, four FBPase-containing peaks were identified. The first peak contained FBPase and the plasma membrane protein Pma1p [54]. The second and third peaks were purified and contained clusters of Vid vesicles of different sizes [44]. The fourth peak was purified and consisted of Vid vesicles, as shown by negative staining and TEM to have diameters of 30–50 nm [65]. The biogenesis of Vid vesicles requires the ubiquitin conjugation enzyme 1 (Ubc1p) and the formation of ubiquitin chains [66]. The sequestration of FBPase into Vid vesicles is dependent on the cytosolic heat shock protein Ssa1p/Ssa2p and the peptidylprolyl isomerase cyclophilin A [43,46]. In addition, COPI coatomer proteins are localized to Vid vesicles as peripheral proteins and are trafficked to endosomes during anterograde trafficking [48]. They are distributed on retrograde vesicles that form on the vacuole membrane in response to glucose re-feeding [48]. Vid24p, Vid28p, and Vid30p are also localized to Vid vesicles and are required for FBPase degradation in the vacuole [67,68,69].

## 3. Gluconeogenic Enzymes Are Secreted into the Periplasm during Glucose Starvation

**Figure 1:**
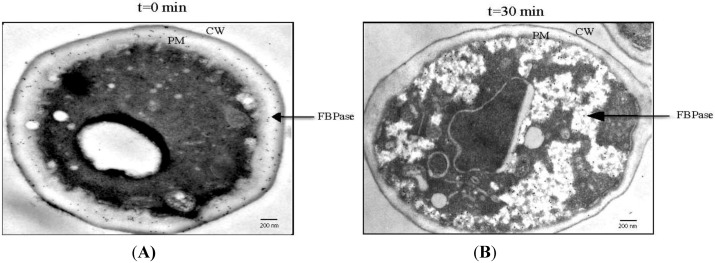
FBPase is re-distributed from the periplasm to the cytoplasm following glucose addition. Wild-type cells were grown in low glucose media for 3 days and harvested (*t* = 0 min) (**A)**; or transferred to high glucose media for 30 min and harvested (*t* = 30 min) (**B**). Cells were processed and FBPase was visualized by immuno-TEM. Bars: 200 nm, PM: plasma membrane, CW: cell wall. This figure is in a manuscript published in Proteome Science [73].

Recent evidence indicates that gluconeogenic enzymes are secreted into the periplasm when yeast cells are starved of glucose for 3 days [70,71,72]. To observe the distribution of gluconeogenic proteins at the ultra-structural level, immuno-TEM was used [71,73]. Wild-type cells were grown in low glucose for 3 days and processed for immuno-TEM. Thin sections of cells were incubated with affinity purified antibodies and then with goat anti-rabbit secondary antibodies conjugated with 10 nm gold particles. In wild-type cells grown in low glucose for 3 days, about 80% of the FBPase was in the periplasm and 20% was in the cytoplasm (Figure 1A). Likewise, significant amounts of MDH2, Icl1p, and Pck1p were in the periplasm in glucose-starved cells [71]. In addition, non-gluconeogenic enzymes such as glyceraldehyde-3-phosphate dehydrogenase (GAPDH) and cyclophilin A (Cpr1p) were distributed in the periplasm in cells grown in low glucose [71]. Quantification of immuno-TEM gold particles indicated that 76.7% of FBPase, 36.9% of MDH2, 46.9% of Icl1p, 57.6% of Pck1p, 33.7% of GAPDH, and 42.7% of Cpr1p were in the periplasm in glucose-starved wild-type cells [71].

The secretion of gluconeogenic enzymes has been reported in multiple secretomic studies [74,75,76,77,78]. For instance, fructose-1,6-bisphosphatase, phosphoenolpyruvate carboxykinase, and malate dehydrogenase are found in the secretome of *Clonorchis sinensis* [78], while fructose-1,6-bisphosphatase, malate dehydrogenase, and isocitrate lyase are detected in the secretome of *Bacillus anthracis* [74]. Malate dehydrogenase is also present in the secretome of *Schistosoma mansoni* and phosphoenolpyruvate carboxykinase is detectable in the secretome of *Schistosoma mansoni* and *Echinostoma caproni* [76]. Additionally, gluconeogenic enzymes are found in extracellular vesicles released from *Histoplasma capsultatum* [79] and are present in exosomes from a mouse insulinoma cell line [80]. Thus, the secretion of gluconeogenic enzymes is widely observed from bacterial to animal cells. Similarly, the secretion of glyceraldehyde-3-phosphate dehydrogenase and cyclophilin A is also observed from yeast grown in high glucose [81,82] and from other organism ranging from bacteria to humans [75,76,77,78,83,84,85,86].

## 4. The Use of an Extraction Protocol to Detect Gluconeogenic Enzymes in the Extracellular Fraction in Glucose-Starved Cells

As shown by immuno-TEM, FBPase, MDH2, Icl1p, Pck1p, GAPDH, and Cpr1p are secreted into the periplasm in glucose-starved cells. In order to obtain extracellular proteins from this location for further analysis, an extraction protocol was used [68,71,72,87]. The method of extraction has previously been utilized to study the secretion of mammalian galectin-1 expressed in *Saccharomyces cerevisiae* [88]. A comparable method has been used to identify proteins associated with the cell wall in *Candida albicans* [89,90]. Proteins that are secreted directly into the media can be collected from the culture media without the use of this method.

To extract gluconeogenic enzymes from the periplasm, cells were incubated in buffer containing the reducing agent β-mercaptoethanol (βME) and Tris with pH 9.4 at 37 °C [91,92]. In control experiments, extracted cells were able to exclude trypan blue and also internalized from the media and actively transported the vital dye FM 4-64 to the vacuole membrane [73]. This indicates that cells are viable and the plasma membrane is intact after the extraction procedure. Furthermore, this protocol detects the presence of known periplasmic proteins such as Scw4p and Scw11p in the extracellular fraction [73]. In contrast, signaling molecules involved in the inactivation and degradation of gluconeogenic enzymes are distributed mostly in the intracellular fraction [72]. Vps34p is the major phosphatidylinositol 3-kinase (PI3K) in yeast and Tor1p is a subunit of the TORC1 complex. Both were detected in the intracellular fraction [73]. Tpk1p, a subunit of the protein kinase A that phosphorylates FBPase [58,59,60] in response to glucose replenishment, was also distributed in the intracellular fraction [73].

Most importantly, the extraction procedure detects the presence of FBPase, MDH2, Icl1p, Pck1p, GAPDH, and Cpr1p in the extracellular fraction in glucose-starved cells [93]. Wild-type cells that expressed tagged proteins were grown in low glucose media for 3 days and then extracted. Proteins that were released into the supernatant following the extraction procedure were precipitated with TCA, washed and then solubilized in SDS sample buffer to form the extracellular (E) fraction. Proteins from the cell-associated fraction were solubilized in SDS sample buffer to form the intracellular (I) fraction. Western blotting was used to determine the distribution of tagged proteins in the intracellular and extracellular fractions. In glucose-starved cells, FBPase, MDH2, Icl1p, Pck1p, GAPDH, and Cpr1p were present in the extracellular fraction [93]. In contrast, Sec28p and signaling molecules involved in the Vid pathway were mainly distributed in the intracellular fraction [72].

As mentioned, the site of FBPase degradation is dependent on the duration of starvation. Having established that the extraction protocol was useful in detecting the presence of FBPase, MDH2, Icl1p, Pck1p, GAPDH, and Cpr1p in the extracellular fraction, this protocol was used to examine whether or not secretion of these proteins into the extracellular fraction was dependent on the duration of starvation. Wild-type cells expressing tagged proteins were starved of glucose for 1 d, 2 d, and 3 d. Cells were then extracted and the distribution of these proteins in the extracellular fraction was determined [93]. Levels of FBPase, MDH2, and GAPDH in the extracellular fraction were low in 1 d-starved wild-type cells and increased in 3 d-starved cells. Conversely, amounts of extracellular Cpr1p were high in 1 d-starved cells and decreased in 3 d-starved cells. Levels of Icl1p and Pck1p in the extracellular fraction were similar in 1 d- *vs.* 3 d-starved cells. Thus, the amounts of FBPase, MDH2, GAPDH, and Cpr1p in the extracellular fraction vary depending on the duration of starvation [93].

## 5. Proteomic Approach to Identify Extracellular Proteins in Glucose-Starved Cells

Now established as an effective method for detecting extracellular proteins present in the periplasm, this extraction protocol was utilized to obtain extracellular proteins for a large-scale proteomic analysis. Wild-type cells were starved of glucose for 3 days and extracted. Extracellular proteins were digested by trypsin and the resulting trypsin fragments were subjected to proteomic analysis [93]. Ninety-two extracellular proteins were identified. As expected, MDH2, Icl1p, Pck1p, GAPDH, and Cpr1p were identified in this study. The extracellular proteins were further classified into multiple functional groups that included enzymes in the metabolisms of carbohydrates, amino acids, nucleotides, and lipids. Heat shock proteins, anti-oxidative proteins and proteins involved in other functions were also identified [93]. Of the 92 extracellular proteins, more than 95% do not contain the classical ER sequences and, as a result, are secreted by the non-classical pathway. Hence, the non-classical pathway is the major pathway to secrete proteins in glucose-starved cells. This conclusion is also observed in a previous proteomic study using yeast cells grown in high glucose [94]. In that study, 99 extracellular proteins were identified and only 17 proteins contained the ER sequence [94]. Many of these extracellular proteins found in yeast have also been identified in multiple large-scale secretomic/proteomic studies from organisms that ranged from bacteria to humans [75,76,77,78,83,84,85,86], suggesting that the secretion of these proteins via the non-classical pathways is conserved across species.

## 6. Vid Vesicles are Secreted as Extracellular Vesicles in Glucose-Starved Cells

**Figure 2:**
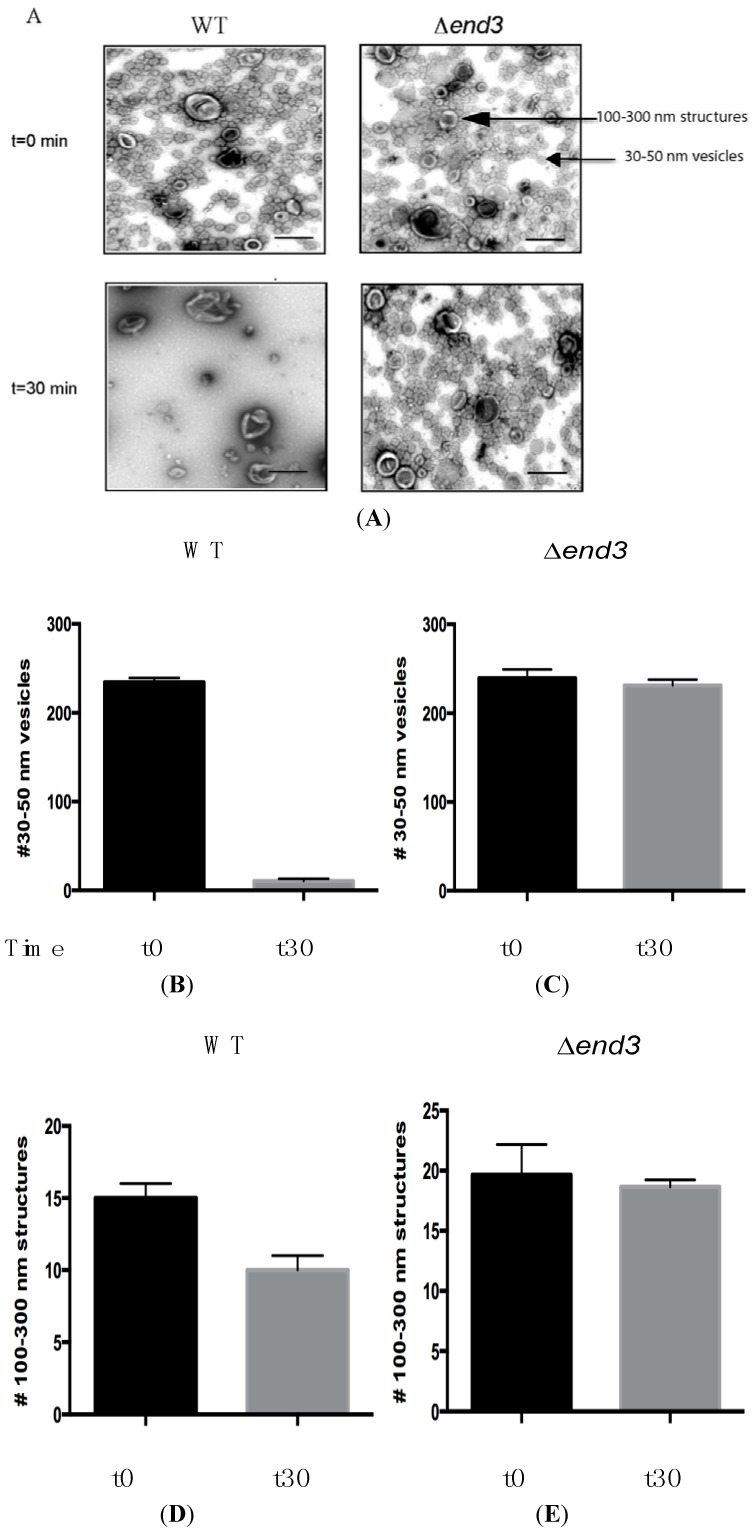
Glucose induces a rapid decline of small vesicles in the extracellular fraction. Wild-type and *∆end3* cells were glucose starved for 3 days and re-fed with glucose for 30 min. Total extracts were obtained and centrifuged at 3000× *g* and then at 200,000× *g*. The 200,000× *g* pellet fraction was fixed, stained with uranyl acetate, and visualized by TEM (**A**). Bars: 200 nm. The number of 30–50 nm small vesicles per µm^2^ at *t* = 0 and *t* = 30 min in wild-type (**B**) and *∆end3* (**C**) cells was quantified. The number of 100–300 nm large structures per µm^2^ at *t* = 0 and *t* = 30 min in wild-type (**D**) and *∆end3* (**E**) cells was quantified. This figure is in a manuscript published in the Journal of Extracellular Vesicles [93].

During glucose starvation, Vid vesicles that carry gluconeogenic enzymes are distributed in multiple locations. Free Vid vesicles are in the cytoplasm and are enriched in the 200,000× *g* pellet fraction [65]. These Vid vesicles can aggregate to form clusters and associate with actin patches in the cytoplasm [48,69]. Interestingly, Vid vesicles are also secreted as extracellular vesicles and enriched in the 200,000× *g* pellet fraction in total extracts [93]. To observe extracellular Vid vesicles, total extracts were obtained from wild-type cells that were grown in low glucose for 3 days. The extract was centrifuged first at 3000× *g* and at 200,000× *g*. The 200,000× *g* pellets were then stained with uranyl acetate and visualized by TEM (Figure 2A). At least two types of structures were observed in total extracts from glucose-starved wild-type cells. Approximately 93%–96% of the extracellular structures consisted of Vid vesicles that were 30–50 nm in diameter, while the remaining 4%–7% were larger structures with diameters ranging from 100 to 300 nm (Figure 2A). As such, extracellular Vid vesicles account for the vast majority of the structures present in total extracts in glucose-starved cells.

## 7. Extracellular FBPase, MDH2, Icl1p, Pck1p, GAPDH, and Cpr1p are Associated with Membranes in the Vesicle-Enriched Fraction in Total Extracts

**Figure 3:**
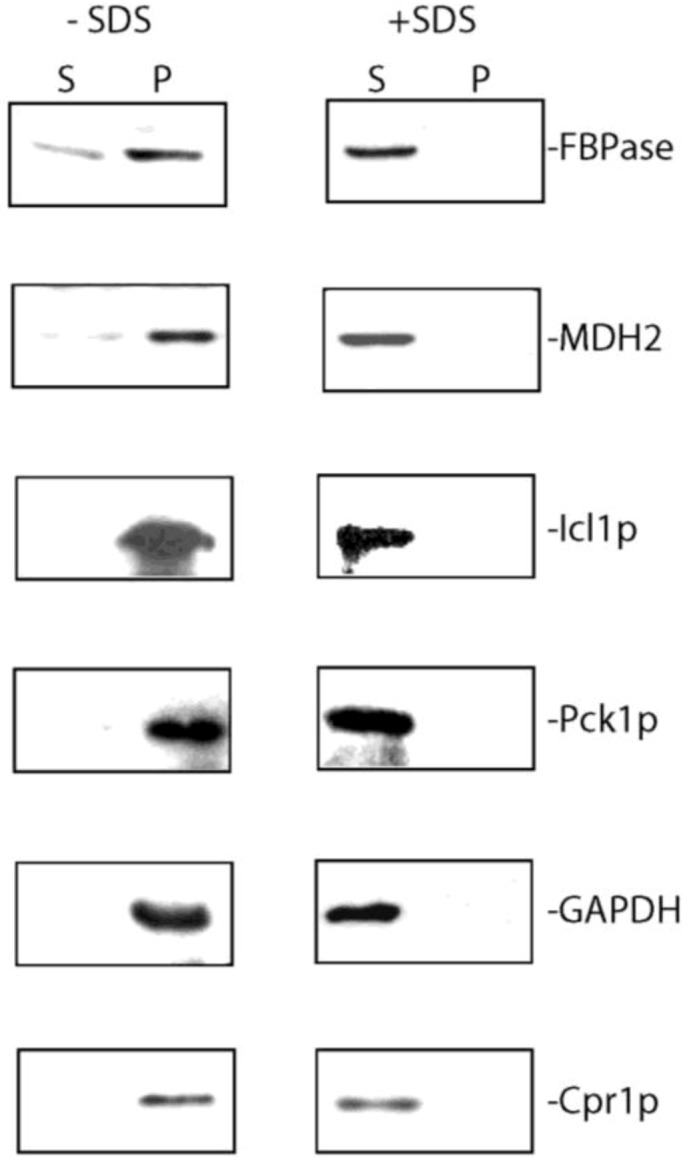
FBPase, MDH2, Icl1p, Pck1p, GAPDH, and Cpr1p are associated with vesicles in the extracellular fraction. Total extracts were obtained from wild-type cells starved of glucose for 3 days and subjected to ultracentrifugation. The final 200,000× *g* pellet fraction was aliquoted, and incubated with or without 2% SDS for 30 min. Samples were re-centrifuged at 200,000× *g* for 2 h following incubation. Western blotting was used to examine the distribution of FBPase, MDH2, Icl1p, Pck1p, GAPDH, and Cpr1p in the 200,000× *g* supernatant (S) and pellet (P) fractions. This figure is in a manuscript published in the Journal of Extracellular Vesicles [93].

In the extracellular fraction of glucose-starved wild-type cells, secreted gluconeogenic enzymes are associated with extracellular Vid vesicles that were enriched in the 200,000× *g* pellet fraction [93]. Wild-type cells expressing tagged proteins were grown in low-glucose media for 3 days and extracted. Total extracts were first centrifuged at 3000× *g* and then at 200,000× *g*. The pellet fraction was resuspended in PBS buffer, aliquoted and incubated in the absence and presence of detergent (2% SDS). Following incubation, samples were re-centrifuged for 2 h at 200,000× *g* and the distribution of proteins in the supernatant (S) and pellet (P) fractions was determined (Figure 3). FBPase, MDH2, Icl1p, Pck1p, GAPDH, and Cpr1p were in the 200,000× *g* pellet fraction in the absence of detergent (Figure 3, left panels). In contrast, these proteins were in the 200,000× *g* supernatant fraction when membranes were solubilized by 2% SDS (Figure 3, right panels). Therefore, membrane integrity is required for the distribution of extracellular gluconeogenic enzymes (FBPase, MDH2, Icl1p and Pck1p) and non-gluconeogenic enzymes (GAPDH and Cpr1p) in the vesicle-enriched fraction.

## 8. Glucose Induces a Rapid Clearance of Vid Vesicles and Vesicle-Associated Proteins from the Extracellular Fraction

In order to observe the effects of glucose in the secretome/extracellular fraction, wild-type cells were starved of glucose for 3 days and harvested (*t* = 0) or transferred to glucose for 30 min and then harvested (*t* = 30). Cells were subsequently extracted and the total extracts were centrifuged at 3000× *g* and then at 200,000× *g*. For visualization using TEM, the 200,000× *g* pellet fraction was suspended in PBS buffer, fixed, and then stained with uranyl acetate (Figure 2). When total extracts were prepared from wild-type cells grown in low glucose (*t* = 0 min), more than 90% of total extracts contained small vesicles and less than 10% were 100–300 nm large structures (Figure 2A, left panels). Following addition of glucose for 30 min (*t* = 30), most of the small vesicles were no longer present, while the 100–300 nm structures were still observed. The number of small vesicles was 234.3 ± 4.7 per µm^2^ in *t* = 0 cells and was 10.7 ± 2.5 per µm^2^ in *t* = 30 cells (Figure 2B). The number of 100–300 nm large structures was 13.5 ± 1.1 per µm^2^ before glucose addition and 10.3 ± 1.2 per µm^2^ after glucose addition (Figure 2D). Hence, the addition of glucose caused a 95.5% reduction in small vesicles while 76.3% of the large structures remained [93].

**Figure 4:**
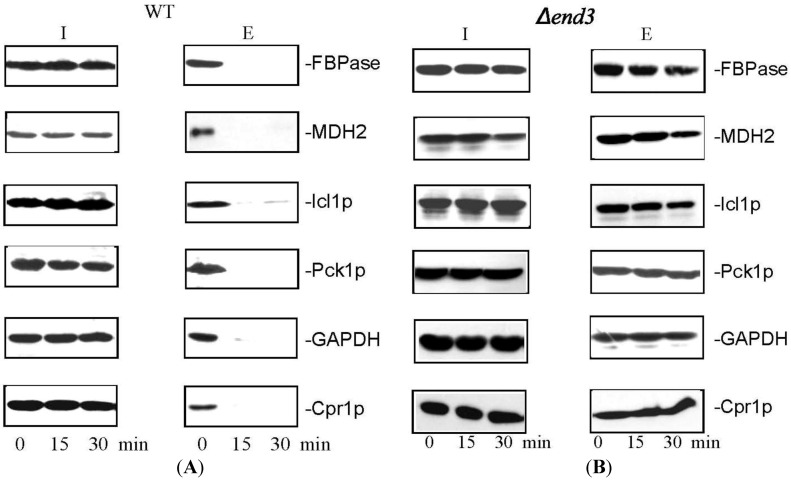
The decline of extracellular FBPase, MDH2, Icl1p, Pck1p, GAPDH, and Cpr1p in response to glucose re-feeding is dependent on *END3*. Wild-type cells (**A**) and the *∆end3* cells (**B**) were starved of glucose for 3 days and re-fed with glucose for 0, 15, and 30 min. Western blotting was used to examine levels of FBPase, MDH2, Icl1p, Pck1p, GAPDH, and Cpr1p in the intracellular (I) and extracellular (E) fractions. This figure is in a manuscript published in the Journal of Extracellular Vesicles [93].

Given that FBPase, MDH2, Icl1p, Pck1p, GAPDH, and Cpr1p are associated with vesicles and that these vesicles disappear from the extracellular fraction following glucose re-feeding, these vesicle-associated proteins should be cleared from the extracellular fraction in response to glucose replenishment. Wild-type cells were starved of glucose for 3 days and transferred to media containing glucose for 0, 15, and 30 min. Cells were extracted and levels of proteins in the extracellular fraction were examined by Western blotting (Figure 4). High levels of FBPase, MDH2, Icl1p, Pck1p, GAPDH, and Cpr1p were observed in the extracellular fraction at *t* = 0 min (Figure 4A). These proteins decreased levels at the *t* = 30 min time point (Figure 4A). Hence, glucose causes a rapid reduction of vesicle-associated proteins in the extracellular fraction.

## 9. FBPase is Re-Distributed from the Periplasm to the Cytoplasm in Response to Glucose

The rapid decline of vesicle-associated proteins in response to glucose addition may result from their internalization into the cytoplasm or release into the culture media. Because FBPase is degraded in the vacuole following glucose addition, secreted FBPase that associates with extracellular vesicles should be internalized after glucose addition prior to being delivered to the vacuole. The distribution of FBPase in response to glucose addition was examined using immuno-TEM [72,73]. Wild-type cells were grown in low glucose media for 3 days and harvested or transferred to glucose-enriched media for 30 min and then harvested. Cells were processed for immuno-TEM. The majority of the FBPase was observed in the periplasm when cells were grown in low glucose for 3 days (Figure 1A, arrow). At the *t* = 30 min time point, most of the FBPase was found in intracellular structures that contained clusters of vesicles (Figure 1B, arrow). Taken together, these results suggest that FBPase and vesicles are internalized in response to glucose addition resulting in the appearance of FBPase and vesicles in the cytoplasm in glucose re-fed cells. To rule out the possibility that small vesicles were released into culture media following glucose addition, culture media was collected from cells that were starved and re-fed with glucose, subjected to ultracentrifugation and followed by observation using TEM. Very few vesicles were observed in the culture media, indicating that glucose does not cause the release of vesicles into the culture media.

## 10. *VPS34* is Required for the Decline of Extracellular FBPase in Response to Glucose

**Figure 5:**
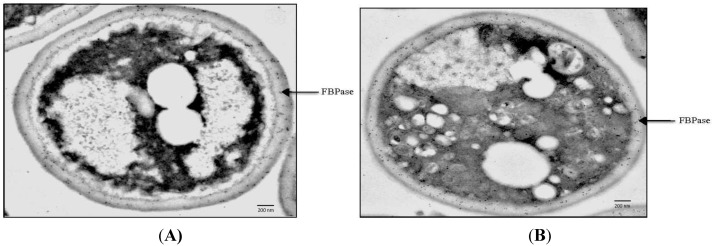
FBPase fails to be internalized in response to glucose in cells lacking the *VPS34* gene. The ∆*vps34* mutant was starved of glucose for 3 days (**A**) and then re-fed with glucose for 2 h (**B**). Cells were processed and examined for the distribution of FBPase using immuno-TEM. This figure is in a manuscript published in the Journal of Biological Chemistry [72].

Internalization of FBPase following glucose addition requires the *VPS34* gene, which encodes the major PI3K in yeast [72]. The role that *VPS34* plays in FBPase distribution was examined using immuno-TEM. The ∆*vps34* mutant was grown in low glucose for 3 days and transferred to glucose. Thin sections of the ∆*vps34* mutant were incubated with affinity purified anti-FBPase and then followed by incubation in goat anti-rabbit secondary antibodies conjugated with 10 nm gold particles. When the ∆*vps34* mutant was starved of glucose, most of the FBPase was in the periplasm while small amounts were in the cytoplasm (Figure 5A, arrow). Following glucose addition to the ∆*vps34* mutant, the majority of FBPase remained in the periplasm (Figure 5B, arrow). To confirm these immuno-TEM results, levels of extracellular FBPase before and after glucose addition were examined with Western blotting [72]. In wild-type cells, levels of extracellular FBPase were high at *t* = 0 min and decreased following glucose addition for 30 min. In the ∆*vps34* mutant, high levels of FBPase were observed in glucose-starved cells and levels did not decrease after glucose addition [72]. Therefore, the *VPS34* gene is required for the decline and internalization of extracellular FBPase in response to glucose addition (Table 1).

The N736 residue and the *C*-terminal 11 amino acids of Vps34p are essential for PI3K activity. When either the N736 residue was mutated or when the *C*-terminal amino acids were deleted, levels of FBPase did not decrease rapidly after glucose addition to glucose-starved mutant cells [72]. Thus, the N736 residue and the *C*-terminal 11 amino acids of Vps34p are critical for the decline of extracellular FBPase in response to glucose addition.

**Table 1:** FBPase distribution in wild-type, ∆*vps34*, and ∆*end3* mutant strains during glucose starvation and following glucose re-feeding.

Strain	Glucose Starvation	Glucose Re-Feeding
**WT**	Secretion	Internalization
∆***vps34***	Normal secretion	Defective internalization
∆***end3***	Normal secretion	Defective internalization

## 11. *END3* is Essential for the Glucose-Induced Decrease of Extracellular Vesicles and Vesicle-Associated Proteins

The endocytosis gene *END3* plays an important role in the decline of extracellular Vid vesicles and vesicle-associated proteins in response to glucose [93]. The ∆*end3* strain was grown in low glucose media and harvested (*t* = 0) or transferred to glucose-rich media for 30 min and then harvested (*t* = 30). Total extracts were centrifuged at 3000× *g* and the resulting supernatant was further centrifuged at 200,000× *g*. The final pellet fraction was re-suspended in buffer, fixed, stained with uranyl acetate, and visualized by TEM (Figure 2A, right panels). Total extracts isolated from ∆*end3* cells contained 239.2 ± 9.7 small vesicles (30–50 nm diameter) per µm^2^ before glucose addition and 231.1 ± 6.6 per µm^2^ after glucose addition (Figure 2C). The number of large structures in total extracts isolated from ∆*end3* cells was 19.7 ± 2.5 per µm^2^ in glucose-starved cells and was 18.6 ± 1.6 per µm^2^ in glucose re-fed cells (Figure 2E). Given that small vesicles were not cleared from the extracellular fraction when glucose was added to glucose-starved ∆*end3* cells, the *END3* gene is critical for the decline of extracellular vesicles in response to glucose replenishment.

Because the *END3* gene is required for the glucose-induced decline of extracellular Vid vesicles, proteins that associate with these extracellular vesicles should also show an *END3*-dependent decrease in levels following glucose addition. Wild-type and the ∆*end3* strain were starved of glucose for 3 days and then transferred to glucose-rich media for 15 and 30 min. Levels of extracellular FBPase, MDH2, Icl1p, Pck1p, GAPDH, and Cpr1p were examined. In glucose-starved wild-type cells, these proteins were present in the extracellular fraction (Figure 4A). Their levels decreased after glucose addition to wild-type cells (Figure 4A). In the ∆*end3* strain, these proteins were also observed in the extracellular fraction at *t* = 0 (Figure 4B). However, levels of these proteins did not decrease following a transfer to glucose for 30 min (Figure 4B). Thus, the *END3* gene is required for the decline and internalization of extracellular vesicles and vesicle-associated proteins in response to glucose addition (Table 1).

Although the molecular mechanisms for the internalization of extracellular vesicles from the periplasm are largely unknown, several features of this endocytosis process suggest that this is a new type of endocytosis. First, it is utilized to transport vesicles from the periplasm to the cytoplasm. This vesicle-mediated transport is distinct from the receptor-mediated process in which ligands bind to their receptors on the plasma membrane before they are internalized. Secondly, this is regulated by changes in glucose concentrations in the environment. It occurs when cells are transferred from low to high glucose. Therefore, this is different from the fluid phase endocytosis, which is constitutive. Thirdly, this is a very high capacity process. At the *t* = 30 min time point, clusters of small vesicles occupy about 10%–20% of the total volume of cytoplasm (Figure 1B). Finally, the endocytosis process is rapidly occurring, as the decline of proteins/vesicles in the extracellular fraction is almost complete within the first 30 min after glucose addition. The rapid clearance of extracellular vesicles and vesicle-associated proteins may enable cells to adjust quickly to the changing environment. Based on these features, this vesicle-mediated endocytosis is fundamentally different from the conventional receptor-mediated endocytosis and the fluid phase endocytosis.

## 12. Conclusions

**Figure 6:**
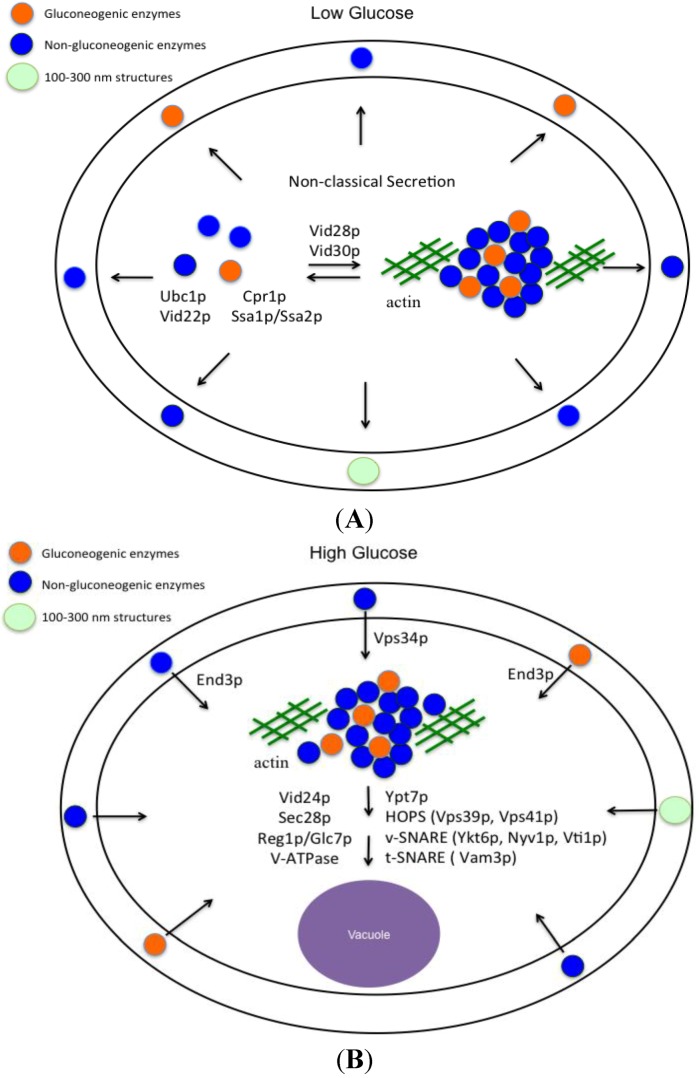
Vid vesicles are secreted into the periplasm during glucose starvation and are internalized following glucose addition. When yeast cells are grown in low glucose, Vid vesicles that carry gluconeogenic enzymes and non-gluconeogenic enzymes are secreted into the periplasm via the non-classical secretory pathway (**A**). In the periplasm, 100–300 nm structures are also present (**A**). In the cytoplasm, FBPase is associated with Vid vesicles. The association is dependent on Ubc1p, Ssa1p/Ssa2p, Vid22p, and Cpr1p. Vid vesicles also aggregate to form large clusters and associate with actin patches. This step requires the *VID28* and *VID30* genes. Following glucose addition to glucose-starved cells for 30 min, FBPase is internalized (**B**). The internalization requires the endocytosis gene *END3* and the *VPS34* gene encoding PI3K (**B**). The 100–300 nm structures decrease in number but are still observed at the *t* = 30 min time point. Following internalization, FBPase is delivered to the vacuole to be degraded, while proteins that are not degraded may be recycled. Vid24p, Sec28p, Reg1p/Glc7p, V-ATPase, Ypt7p, HOPS, v-SNARE and t-SNARE are critical for the post-internalization step in the Vid pathway [44,48,98,99,100].

The Vid pathway that was originally described for the trafficking of gluconeogenic enzymes to the vacuole for degradation can be divided into three major parts: (a) secretion of gluconeogenic and non-gluconeogenic enzymes in vesicles into the periplasm during glucose starvation (Figure 6A); (b) internalization of these proteins following glucose addition; and (c) trafficking of vesicles to the vacuole (Figure 6B). In glucose-starved cells, these proteins are secreted via the non-classical pathway and are associated with Vid vesicles in the extracellular fraction. Vid vesicles exist as free form and aggregated form in the cytoplasm. The biogenesis of Vid vesicles is dependent on Ubc1p (ubiquitin conjugating enzyme 1) [66], whereas the association of FBPase with Vid vesicles requires the cytosolic 70 kD heat shock protein Ssa1p/Ssa2p [46], Vid22p [47], and Cpr1p [43]. These Vid vesicles also aggregate to form large clusters and associate with actin patches. This step requires Vid28p and Vid30p [68,95]. It remains to be determined whether or not extracellular vesicles are released from the intracellular free vesicles or from aggregated vesicles in the cytoplasm. In cells grown in low glucose, 100–300 nm structures are also distributed in the extracellular fraction (Figure 6A). Using a large-scale proteomic approach, 92 proteins that were present in the extracellular fraction in glucose-starved cells were identified. More than 95% of these extracellular proteins do not contain the typical signal sequence for the ER-Golgi pathway, suggesting that they are secreted via the non-classical pathway. Therefore, the non-classical pathway is the major pathway to secrete proteins into the extracellular fraction. This conclusion is observed in cells grown in low glucose as well as in cells grown in high glucose.

Interestingly, secreted gluconeogenic enzymes and non-gluconeogenic enzymes are associated with extracellular Vid vesicles in glucose-starved cells (Figure 6A). These extracellular vesicles account for more than 90% of the total structures present in total extracts. Vid vesicles are 30–50 nm in diameter, have densities of 1.2 g/mL [65], and are associated with secreted gluconeogenic enzymes in the total extracts. Exosomes with a diameter of 40–100 nm and a density of 1.1–1.2 g/mL are secreted from a variety of mammalian cells [95,96,97]. Gluconeogenic enzymes FBPase and MDH2 have also been identified in exosomes purified from the mouse insulinoma NIT-1 cells [80]. Thus, extracellular Vid vesicles that carry gluconeogenic enzymes in yeast cells share features similar to those observed in exosomes secreted from mammalian cells. Secreted gluconeogenic enzymes that associate with extracellular vesicles during glucose starvation are internalized into the cytoplasm after glucose addition. This internalization is dependent on the *VPS34* gene and the *END3* gene (Figure 6B). Internalization results in a decline in extracellular gluconeogenic enzymes and the disappearance of small vesicles from the extracellular fraction at the *t* = 30 time point. Moreover, internalization also leads to the appearance of clusters of FBPase and small vesicles in the cytoplasm [44,72]. Vid/endosomes have been purified to near homogeneity from the cytoplasm of wild-type cells re-fed with glucose [44]. Inside these Vid/endosomes, FBPase was associated with small vesicles [44], indicating that FBPase is internalized in these vesicles. Internalization is also consistent with the findings that the endocytosis gene *END3* is required for the decline of extracellular vesicles and vesicle-associated proteins including FBPase, MDH2, Icl1p, Pck1p, GAPDH, and Cpr1p in response to glucose addition [93]. Following internalization, FBPase is delivered to the vacuole for degradation. This post-internalization step requires Vid24p, Sec28p (COPI coatomer subunit)*,* Reg1p/Glc7p phosphatase, the vacuole-ATPase, HOPS (homtypic fusion vacuole protein sorting complex Vps39p and Vps41p), v-SNARE (Ykt6p, Nyv1p, Vti1p), and *t*-SNARE (Vam3p) [44,48,98,99,100].

As mentioned above, Vid vesicles share many similar physical properties with extracellular vesicles (EVs) or exosomes released from the mammalian cells. However, the biogenesis, secretion and internalization of Vid vesicles may not be the same as those described for EVs. The formation, exocytosis, and endocytosis of EVs have been reviewed extensively by several investigators [101,102,103,104,105,106,107]. Readers are encouraged to view these articles for a complete understanding of these processes. Briefly, EVs are produced by inward budding of MVBs (multivesicular bodies) using the ESCRT complex. Rabs proteins are required for MVBs trafficking to the plasma membrane. Following the v-SNARE and t-SNARE pairing, MVBs then fuse with the plasma membrane to release EVs into the extracellular space. Because EVs are produced by inward budding of the MVB membranes, these vesicles should not be present in the cytoplasm. For the Vid pathway, free Vid vesicles are present in the cytoplasm constitutively. Likewise, clusters of Vid vesicles are also present in the cytoplasm before and after glucose addition. Furthermore, MVBs are enclosed by a single layer of outer membrane, whereas an outer membrane was not observed in clusters of Vid vesicles. However, both Vid vesicle clusters and MVBs contain numerous small vesicles within these organelles.

For endocytosis of EVs, different mechanisms such as fusion, clathrin-mediated endocytosis, and macropinocytosis have been implicated [102,103,108]. During heterotypic membrane fusion, the donor membrane and acceptor membrane fuse which results in the integration of these two different membranes into one membrane. If EVs fuse with the plasma membrane, EVs’ membranes become part of the plasma membrane, whereas the internal contents from EVs will be released directly into the cytoplasm. However, EVs labeled with fluorescent lipid dye were observed using fluorescence microscopy to be in the cytoplasm in acceptor cells as punctate structures [108]. This suggests that the exosome membranes are not integrated into the plasma membrane. Therefore, fusion is unlikely to explain the uptake of EVs into acceptor cells. Likewise, fusion is unlikely to explain the internalization of FBPase following glucose addition. If extracellular Vid vesicles fuse with the plasma membrane, then FBPase should be distributed near the plasma membrane or as free protein in the cytoplasm. Because FBPase was distributed in clusters of vesicles in the cytoplasm following internalization, fusion of extracellular Vid vesicles with the plasma membrane should not happen. It remains to be determined whether or not clathrin-mediated endocytosis is used for FBPase uptake into the cells.

At the present time, the molecular mechanisms required for Vid vesicle trafficking in and out of the cell are not well understood. In the future, it will be important to purify the 100–300 nm structures and determine whether or not these structures are involved in the secretion or internalization of vesicles. It will also be imperative to identify genes required for the biogenesis and trafficking of Vid vesicles across the plasma membrane.
